# Clinical Insights into Vestibular Disorders

**DOI:** 10.3390/jcm13237375

**Published:** 2024-12-04

**Authors:** Eduardo Martin-Sanz

**Affiliations:** 1 Department of Otolaryngology, University Hospital of Getafe, 28095 Madrid, Spain; emartinsanz@gmail.com; 2 Department of Medicine, School of Biomedical Sciences and Health, Universidad Europea de Madrid, 28670 Villaviciosa de Odón, Spain

We are pleased to present a Special Issue addressing the current challenges in vestibular disorders.

This Special Issue comprises six publications covering various topics related to the diagnosis and treatment of vestibular disorders.

Recent clinical insights into vestibular disorders have clarified conditions that impact balance, coordination, and spatial orientation, fundamentally influencing patients’ quality of life.

Disorders like benign paroxysmal positional vertigo (BPPV), genetic hearing loss, vestibular schwannoma, visual vertigo, persistent postural perceptual dizziness (PPPD) and diverse neurological conditions may affect the vestibular system, a key component of the inner ear responsible for balance and spatial awareness. Advances in both diagnosis and treatment are beginning to offer hope for more accurate and individualized management of these conditions, which have historically been difficult to diagnose and treat.

Relatively new clinical scenarios warrant consideration in the assessment of vestibular disorders. For example, the vestibular phenotypes of patients with genetic hearing loss remain inadequately understood. In the study by Ji-Hyuk Han et al. [1], a substantial proportion of patients with genetic hearing loss exhibited concomitant vestibular symptoms, which were often overlooked.

Consideration should also be given to less common variants of one of the most prevalent vertigo disorders, such as BPPV. For example, Jun Beom An and colleagues [2] provide a characterization of a pediatric BPPV population and conduct a comparative analysis with an adult BPPV cohort.

Similarly, patients with PPPD frequently experience visual vertigo, making it important for clinicians to monitor changes in their dizziness symptoms over time. Tsun-Ai Jasper Chen and colleagues [3] developed a computerized Visual Vertigo Analogue to assess visual vertigo in individuals with PPPD.

The therapeutic landscape for vestibular disorders is also evolving. For example, BPPV is effectively treated with canalith repositioning maneuvers in the vast majority of patients. However, patients with many clinical conditions face challenges with these maneuvers, potentially experiencing longer healing times and creating additional difficulties for physicians diagnosing and treating BPPV in everyday practice. The emergence of mechanical rotational chairs (MRCs) offers a more convenient alternative for performing these maneuvers. Chaure-Cordero et al. [4] ’s study provides an analysis of both types of canalith repositioning maneuvers.

Treatment options for other vestibular disorders are more limited; thus, new therapies are a welcome development. Vestibular rehabilitation therapy (VRT), which involves specific head, eye, and body movements to retrain the brain’s response to vestibular input, has proven highly effective in improving symptoms and quality of life.

These developments introduce diverse alternatives for more personalized therapeutic approaches to address vertigo and postural control disorders.

In this way, Virginia Fancello et al. [5] evaluate the effectiveness of their intensive customized VRT after vestibular schwannoma excision, and Tina Yu-Zhou Li et al. [6] propose a promising Rehabilitation Oculomotor Screening Evaluation for Acquired Brain Injuries.

## Conclusions

The field of vestibular disorders is undergoing a transformation as new clinical insights improve our understanding of these complex conditions. Enhanced diagnostic tools, advanced knowledge of pathophysiology, and innovative therapies are all contributing to more effective management strategies.

However, many challenges remain. Vestibular disorders are often underdiagnosed, and there is a need for ongoing research to refine treatments further. By building on these recent advances, we move closer to personalized and comprehensive care for vestibular patients, improving both diagnostic accuracy and therapeutic outcomes.
